# Financial and Mental Health Concerns of Impoverished Urban-Dwelling Bangladeshi People During COVID-19

**DOI:** 10.3389/fpsyg.2021.663687

**Published:** 2021-08-06

**Authors:** Md. Saiful Islam, Md. Estiar Rahman, Rajon Banik, Md. Galib Ishraq Emran, Noshin Saiara, Sahadat Hossain, M. Tasdik Hasan, Md. Tajuddin Sikder, Lee Smith, Marc N. Potenza

**Affiliations:** ^1^Department of Public Health and Informatics, Jahangirnagar University, Dhaka, Bangladesh; ^2^Center for Advanced Research Excellence in Public Health, Dhaka, Bangladesh; ^3^Department of Environmental Sciences, Jahangirnagar University, Dhaka, Bangladesh; ^4^Department of Biotechnology and Genetic Engineering, Jahangirnagar University, Dhaka, Bangladesh; ^5^Department of Primary Care and Mental Health, University of Liverpool, Liverpool, United Kingdom; ^6^The Cambridge Center for Sport and Exercise Sciences, Anglia Ruskin University, Cambridge, United Kingdom; ^7^Department of Psychiatry and Child Study Center, Yale School of Medicine, New Haven, CT, United States; ^8^Connecticut Mental Health Center, New Haven, CT, United States; ^9^Connecticut Council on Problem Gambling, Wethersfield, CT, United States; ^10^Department of Neuroscience, Yale University, New Haven, CT, United States

**Keywords:** COVID-19 pandemic, poverty, Bangladesh, sleep, depression, post-traumatic stress disorder

## Abstract

**Background:** The COVID-19 pandemic has impacted the physical, mental and financial health of many individuals. Individuals living in impoverished crowded settings may be particularly vulnerable to COVID-19-related stressors. How substantially marginalized groups like impoverished urban-dwelling individuals have been impacted during this pandemic is poorly understood. The present study aimed to investigate the associated factors of financial concerns and symptoms of depression and posttraumatic stress disorder (PTSD) during the COVID-19 pandemic among impoverished urban-dwelling individuals residing in Dhaka, Bangladesh.

**Methods:** A cross-sectional survey was conducted between August and September 2020 using face-to-face interviews in six disadvantaged neighborhoods (“slums”) in Dhaka. Individuals were interviewed using a semi-structured questionnaire consisting of questions assessing socio-demographics, lifestyle, financial well-being relating to the COVID-19 pandemic, depression, and PTSD.

**Results:** Four-hundred-and-thirty-five individuals (male = 54.7%; mean age = 45.0 ± 12.0 years; age range = 18–85 years) participated. Most (96.3%) reported that their household income decreased due to the COVID-19 pandemic. Factors associated with decreased household incomes included female gender, primary education, joblessness, food scarcity and depression. Depression symptoms were linked to female gender, joblessness, divorce, living in a joint family, excessive sleep and smoking. Low incomes, excessive sleep, joblessness and food scarcity were positively associated with PTSD symptoms. In contrast, less sleep appeared protective against PTSD.

**Conclusions:** Public health initiatives, in particular mental health services that target stress and biocentric approaches that consider how humans interact with multiple facets of nature, should be introduced to mitigate against potential financial and psychological effects of the pandemic on impoverished urban-dwelling individuals in Bangladesh.

## Introduction

The outbreak of the 2019 novel coronavirus (SARS-CoV-2) emerged in China at the end of 2019, and the virus rapidly spread globally (Wang et al., 2020a; Xiang et al., 2020). COVID-19 is considered a new public health crisis and on March 11, 2020, a pandemic was declared by the World Health Organization (WHO) (Cucinotta and Vanelli, 2020). In Bangladesh, the first case of COVID-19 was officially recorded on March 8, 2020 (Banik et al., 2020a; Ferdous et al., 2020). Since then, the total number of confirmed cases has increased swiftly: ~404,760 cases had been confirmed with a death toll of 5,886 as of October 30, 2020 (Institute of Epidemiology Disease Control and Research, 2020). To deal with the pandemic, the governments of most countries have taken unprecedented preventative measures, including nationwide lockdowns, spatial distancing, business and work limitations and other actions (Anderson et al., 2020; Brooks et al., 2020).

In order to limit the spread of COVID-19, the government of Bangladesh declared nationwide restrictions on public activities and movement across the country in March, 2020 (Rahman et al., 2020a; The Daily Star, 2020; Islam et al., 2021a). While these pandemic-related constraints were critical for preventing COVID-19, they also negatively impacted occupational opportunities, increased insecurity and generated financial challenges (Bhuiyan et al., 2020; Galicki, 2020). Pandemic issues such as spatial distancing, isolation, and quarantine, as well as social and economic consequences, have led to anger, boredom, fear, frustration, grief, depression, fear, grief, posttraumatic stress disorder (PTSD), shame, and stress (Brooks et al., 2020; Islam et al., 2020d,f; Tasnim et al., 2021). These constitute common mental health problems that many individuals have been experiencing during the pandemic, and these may continue after the crisis (Banerjee, 2020). Experiencing or witnessing suffering related to COVID-19 may lead to PTSD among survivors, their families, frontline workers, and the general public (Xiao et al., 2020). The COVID-19 pandemic has impacted mental wellbeing disproportionately among specific groups including adolescents, students, women, and healthcare workers, among others (Biviá-Roig et al., 2020; Commodari and La Rosa, 2020; Wang and Zhao, 2020; Tasnim et al., 2021). Moreover, increases in drinking behaviors, problematic use of smartphone, internet, social media, gaming, and other addictive behaviors have been reported during the pandemic (Higuchi et al., 2020; Islam et al., 2020e, 2021e; Rodriguez et al., 2020; La Rosa et al., 2021). These addictive behaviors may be linked to pandemic-related traumatic events (e.g., lockdowns) and have been associated with mental health concerns including anxiety and depression (Higuchi et al., 2020; Rodriguez et al., 2020; Islam et al., 2021e; La Rosa et al., 2021).

The pandemic that spread worldwide in 2020 has detrimentally impacted both human health and the environment (De Vido, 2020). It has been suggested that COVID-19 emerged as a result of humans living in an anthropocentric manner, with humans at the top of the hierarchy. To address current challenges, alternate approaches (e.g., biocentric rather than anthropocentric) to city planning and growth are important to consider in response to and recovery from COVID-19 (de Leeuw, 2020). Stueck (2021) concluded that six biocentric fields of action are needed to maintain humans' relations to themselves, other people, and other living beings in nature during and after pandemics: (i) maintaining effective communication, (ii) maintaining lively corporeality, (iii) interacting with one's own identity and inner-centered self-reflection in collaboration with others, (iv) building life sense and expressing life potentials, (v) expanding consciousness and perceptions of wholeness, (vi) growing ecological understanding and sustainable biocentric lifestyles and attitudes (Stueck, 2021). Such models include considering how people interact with themselves, others and organisms within environments, including cities, and more rural areas. Among groups who may be disproportionately affected by COVID-19 are people living in cities, particularly cities that have faced considerable inequities (de Leeuw, 2020).

The COVID-19 pandemic has impacted individuals globally, and especially impoverished urban-dwelling individuals living within congested environments and with limited resources. Living in such settings may lead to increased transmissibility of the virus and stress. Currently, Dhaka (where the present study was conducted), the capital city of Bangladesh, has more than 3,300 disadvantaged neighborhoods (“slums”) that house around 646,000 people; of these, most are poor day laborers and rickshaw drivers (The Daily Star, 2019; Kamruzzaman, 2020). These blighted areas are densely populated; ~75% of households live in one room [Bangladesh Bureau of Statistics (BBS) and UNICEF Bangladesh, 2014]. In these areas of Bangladesh, population density is very high, estimated at 205,415 individuals/km^2^ (United Nations, 2015; Islam and Kibria, 2020). About 37% of disadvantaged households in urban areas have 26–50 square feet per person [Centre for Urban Studies (CUS) et al., 2006]. In such circumstances, the impoverished urban residents in Dhaka often find themselves in particularly vulnerable conditions (Banik et al., 2020b), in part relating to low levels of income and high levels of financial uncertainty (Bhuiyan et al., 2020). Indeed, a recent report indicated extreme economic fallout due to the COVID-19 crisis among impoverished urban residents in Bangladesh with a reduction in per capita income by 82% from 108 Bangladeshi Taka (BDT) (US$1.30) in February, 2020, to 27 BDT (US$0.32) during the survey week in early April, 2020 (Kamruzzaman, 2020). Therefore, these individuals may be particularly vulnerable to psychological concerns due to extreme levels of financial insecurity exacerbated by the COVID-19 pandemic. A recent study suggested that the most vulnerable and poorest groups in Bangladesh would likely experience socioeconomic crises and substantial mental stress due to the COVID-19 pandemic (Bodrud-Doza et al., 2020; Shammi et al., 2020). Evidence from previous studies also found that the overall negative impact of COVID-19 on the economy, daily life and social activity was associated with greater psychological difficulties (Cao et al., 2020; Zhang and Ma, 2020). Many studies from Bangladesh and China during the initial phases of the COVID-19 pandemic revealed associations between COVID-19-related experiences and anxiety, depression, and posttraumatic stress (Boyraz and Legros, 2020; Cao et al., 2020; Islam et al., 2020f; Liang et al., 2020; Wang et al., 2020b; Zhang and Ma, 2020). Earlier studies conducted in Bangladesh during the COVID-19 pandemic reported that depression was associated with female gender, older age, married status, lower education, large family size (≥5 members), lower family income, urban residence, tobacco smoking, and sleep disturbances (Islam et al., 2020f, 2021b,f; Tasnim et al., 2021), while stress or PTSD was associated with female gender, older age, urban residence, tobacco smoking, and sleep disturbances (Islam et al., 2020f; Zubayer et al., 2020; Sultana et al., 2021).

Studies of general (community-dwelling) people, university students, medical students, healthcare workers and COVID-19 survivors have highlighted various mental health problems in Bangladesh during the pandemic [for instance, anxiety, depression, panic, stress, suicidal ideation, and behavioral problems (like problematic use of smartphone, internet, social media)] (Banna et al., 2020; Islam et al., 2020b,c,e,f, 2021b,c,e; Tasnim et al., 2020, 2021; Safa et al., 2021). However, impoverished urban residents have not been adequately studied. Thus, there is an urgent need to understand the possible psychological issues that are faced by impoverished urban residents during this pandemic. However, to the best of our knowledge, no prior study has investigated psychological measures during the COVID-19 pandemic using any standard psychometric tools among impoverished urban residents in Bangladesh. Consequently, the current study aimed to explore the associated factors of financial poverty and symptoms of depression and PTSD during the COVID-19 outbreak among impoverished urban residents of Dhaka, Bangladesh.

## Materials and Methods

### Study Design and Setting

The present study used a cross-sectional and interview-based survey of impoverished urban residents in Dhaka. The survey was conducted using a structured questionnaire between August and September 2020. The survey included 6 disadvantaged neighborhoods (Aziz Shaheber Bosti Bari, Balur Maath Songlongno Bosti, Fighter Bosti, Khurshid Bari Bosti, Pinur Bosti, and Shorgochera Bosti) located in Dhaka, Bangladesh.

### Study Procedure

All procedures of the present study were conducted in accordance with ethical principles of human investigations (i.e., Helsinki Declaration) and with the guideline of Institutional research ethics. After obtaining the formal ethics approval and the necessary coordination of the ethical review board of Jahangirnagar University [Ref. No: BBEC, JU/ M 2020/COVID-19/(8)5], the present study was initiated. A Bangla questionnaire incorporating informed consent and including questions and measures was employed to conduct face-to-face interviews to gather information from participants while maintaining proper precautions and spatial distancing during the COVID-19 pandemic. Considering the health risks associated with COVID-19, precautionary safety measures were taken during data collection. Participants were informed about the procedures and purpose of the study, and the confidentiality of information they provided. All data were collected anonymously and analyzed using a pre-determined coding system.

### Sampling Method

The sample size was calculated using the following equation:

**Table:** 

$n=\frac{z^{2}pq}{d^{2}};$ $n\;=\;\frac{1.96^{2}\times 0.5\times \left(1-0.5\right)}{0.05^{2}}$ = 384.16≈384	Here, *n* = number of samples *z* = 1.96 (95% confidence level) *p* = prevalence estimate (0.5) *q* = (1-*p*) *d* = precision limit or proportion of sampling error (0.05)

**Figure 1:**
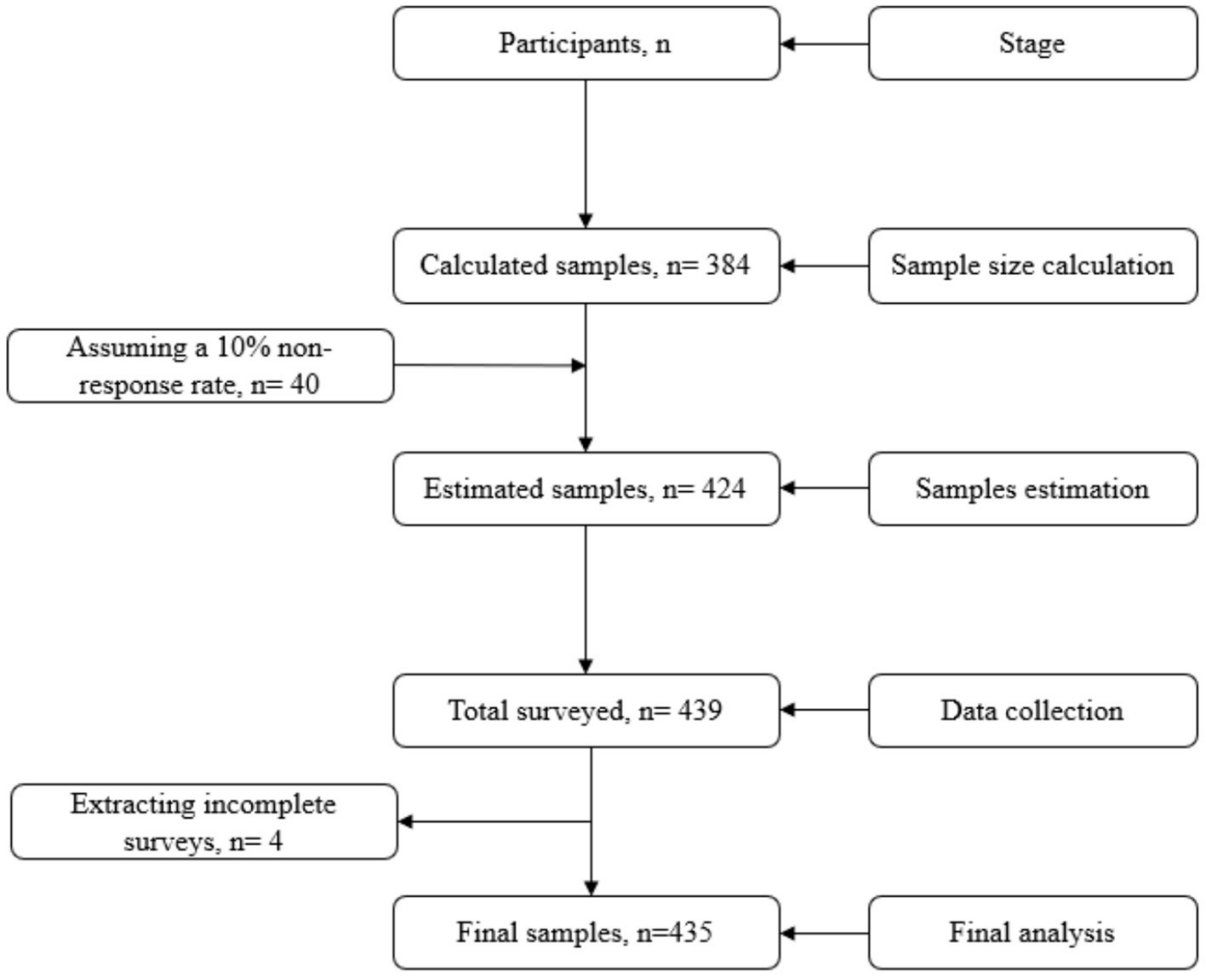
Inclusion of the participants.

There is no prior similar study focusing on the study group during the COVID-19 pandemic. Thus, we hypothesized that psychological problems would be ~50% among impoverished urban residents during the pandemic. Assuming a 10% non-response rate, a total of 423.5 ≈ 424 participants was estimated. However, 435 participants were recruited to ensure adequate power for the study (see Figure 1).

The inclusion criteria for participants were (1) being aged ≥18 years, (2) being impoverished urban residents, and (3) willingness to enroll in the survey. Exclusion criteria were (4) being <18 years old, (5) not being able to provide consent, and (6) having incomplete surveys. After obtaining informed consent, 439 participants were interviewed using a convenience sampling approach. At the data quality checking stage, 4 participants with incomplete surveys were removed, and 435 participants were included in the final analysis.

### Measures

The questionnaire included informed consent and questions assessing socio-demographic, lifestyle, financial and COVID-19-related domains, and depression and PTSD.

#### Socio-Demographics and Lifestyle-Related Measures

Data were collected on gender (male/female), age, marital status (unmarried/married/divorced or widowed), education (no formal education, primary level [1-5 grades], and secondary level [6–10 grades] or greater), occupation, family type (nuclear/joint), and monthly family income. Age was subsequently categorized into two groups: 18–40 years and > 40 years; and monthly family income was categorized into two groups: ≤ 10,000 BDT and > 10,000 BDT.

In addition, numbers of average sleep hours and smoking status were assessed. Average hours of sleep were classified as normal (7–9 h), less than normal (<7h), or more than normal (> 9h) based on previous reports (Hirshkowitz et al., 2015; Chen et al., 2020; Islam et al., 2020a).

#### Financial Measures

The financial impact of COVID-19 was assessed by asking, “*How has your family's monthly income changed due to the impact of COVID-19?.”* There were three possible responses (i) decreased; (ii) increased; (iii) unchanged, as previously reported (Tran et al., 2020). As no participants reported their income “increased” due to the impact of the COVID-19 pandemic, decreased or unchanged incomes were ultimately categorized. Two additional “yes/no” questions were asked during the survey concerning familial job loss and food scarcity due to COVID-19.

#### Patient Health Questionnaire (PHQ-9)

The PHQ-9 is a unidimensional psychometric instrument developed by Spitzer et al. (1999) for assessing depressive disorder. It is a psychometrically sound and robust screening tool used globally in epidemiological surveys. The scale contains nine questions querying about depressive symptoms over the past 2 weeks (e.g., “*Trouble falling or staying asleep, or sleeping too much”*). Responses were assessed with a four-point Likert scale ranging from 0 (*Not at all*) to 3 (*Nearly every day*). The present study utilized the Bangla version of the PHQ-9 (Chowdhury et al., 2004) to assess participants' depressive symptomatology as previously in Bangladeshi samples (Islam et al., 2020b, 2021d; Moonajilin et al., 2020; Rahman et al., 2020b). The total score ranged from 0 to 27, with higher scores reflecting greater severity. In the present study, the reliability coefficient Cronbach's α of the PHQ-9 scale was 0.83.

#### National Stressful Events Survey for PTSD-Short Scale (NSESSS-PTSD)

The NSESSS-PTSD is a brief, easy to use, psychometrically sound and robust instrument for assessing PTSD, developed by LeBeau et al. (2014) and based on *DSM-5* diagnostic criteria. This scale consists of nine-item questions regarding problems related to PTSD symptoms over the last week (i.e., “*Feeling very emotionally upset when something reminded you of a stressful experience”*) with a five-point Likert scale ranging from 0 (*Not at all*) to 4 (*Extremely*). The present study used the Bangla version of the NSESSS-PTSD to assess participants' PTSD. The NSESSS-PTSD was translated following back translation, the most widely used standardized translation, as proposed by Beaton et al. (2000). The total score ranged from 0 to 36, with higher scores reflecting more severe PTSD. In the present study, the internal consistency for the NSESSS-PTSD (Cronbach's α = 0.70) was acceptable (Taber, 2018).

### Statistical Analyses

Analyses were performed using three statistical software packages (Microsoft Excel 2019, IBM SPSS Statistics version 25, and STATA version 13). Microsoft Excel was used to perform data cleaning, coding, editing and sorting. Then an excel file including all variables was imported into SPSS software. For categorical variables, frequencies and percentages were reported; means and standard deviations were presented for continuous data. In addition, some first-order analyses (e.g., Chi-square tests, Fisher's exact tests) were performed using SPSS. Finally, the multiple linear regression model was investigated using STATA to determine the associated factors of depression and PTSD symptomatology. A *p*-value ≤ 0.05 was considered as statistically significant.

## Results

### General Characteristics

Participants (*n* = 435) had a mean age of 45.0 years (SD = 12), and ages ranged from 18 to 85 years. Of participants, 54.7% were male and most were married (87.4%; see Table 1). Most participants had a primary level of education (grades 1–5; 74.3%), had monthly family income ≤ 10,000 BDT (55.9%), and belonged to a nuclear family (89.0%; Table 1). Many indicated that they kept small shops (24.8%). Most reported that they slept in a normal range (7–9 h/day; 66.7%), and a sizeable minority smoked cigarettes (23.2%). A vast majority reported that they had lost their jobs due to the impact of the COVID-19 pandemic (95.6%). Likewise, 98.9% reported they had suffered from food scarcity due to the COVID-19 pandemic.

**Table 1:** Measures and their associations with household income decreases due to the impact of COVID-19.

**Characteristics**	**Household income changes due to COVID-19**				**Total**		**χ^2^**	**df**	***p*** **-value**
	**Yes**		**No**						
	***n***	**(%)**	***n***	**(%)**	***n***	**(%)**			
**Total**	419	(96.3)	16	(3.7)	435	(100)			
**Gender**									
Male	225	(94.5)	13	(5.5)	238	(54.7)	4.72^*^	1	0.039
Female	194	(98.5)	3	(1.5)	197	(45.3)			
**Education**									
No formal education	51	(91.1)	5	(8.9)	56	(12.9)	8.12^*^	2	0.010
Primary level (1–5 grades)	316	(97.8)	7	(2.2)	323	(74.3)			
Secondary level (6–10 grades)	52	(92.9)	4	(7.1)	56	(12.9)			
**Occupation**									
Housewife	53	(100.0)	0	(0)	53	(12.2)	9.45^*^	6	0.087
Workers	94	(96.9)	3	(3.1)	97	(22.3)			
Day laborer	24	(92.3)	2	(7.7)	26	(6.0)			
Rickshaw puller	92	(96.8)	3	(3.2)	95	(21.8)			
Jobless	25	(96.2)	1	(3.8)	26	(6.0)			
Small shop keeping	105	(97.2)	3	(2.8)	108	(24.8)			
Others	26	(86.7)	4	(13.3)	30	(6.9)			
**Marital status**									
Married	367	(96.6)	13	(3.4)	380	(87.4)	2.57^*^	2	0.277
Unmarried	18	(90.0)	2	(10.0)	20	(4.6)			
Divorced	34	(97.1)	1	(2.9)	35	(8.0)			
**Family type**									
Nuclear	373	(96.4)	14	(3.6)	387	(89.0)	0.04^*^	1	0.693
Joint	46	(95.8)	2	(4.2)	48	(11.0)			
**Monthly family income**									
≤ 10,000 BDT	234	(96.3)	9	(3.7)	243	(55.9)	0.01	1	0.975
>10,000 BDT	185	(96.4)	7	(3.6)	192	(44.1)			
**Sleep status**									
<7 h	136	(97.8)	3	(2.2)	139	(32.0)	3.99^*^	2	0.135
7–9 h	278	(95.9)	12	(4.1)	290	(66.7)			
>9 h	5	(83.3)	1	(16.7)	6	(1.4)			
**Tobacco smoking**									
Yes	99	(98.0)	2	(2.0)	101	(23.2)	1.07^*^	1	0.382
No	320	(95.8)	14	(4.2)	334	(76.8)			
**Job loss due to COVID-19**									
Yes	417	(97.0)	13	(3.0)	416	(95.6)	28.74^*^	1	<0.001
No	2	(40.0)	3	(60.0)	19	(4.4)			
**Experiencing food scarcity due to COVID-19**									
Yes	405	(97.4)	11	(2.6)	430	(98.9)	45.29	1	<0.001
No	14	(73.7)	5	(26.3)	5	(1.1)			
	***Mean***	***(SD)***	***Mean***	***(SD)***	***Mean***	***(SD)***	***t***	**df**	***p*** **-value**
**Age**	44.9	(11.9)	45.7	(14.0)	45.0	12.0	0.06	1	0.803
**Depression**	7.2	(4.4)	5.0	(5.0)	7.1	(4.4)	3.86	1	0.050
**PTSD**	15.2	(3.2)	13.7	(7.2)	15.1	(3.4)	3.06	1	0.081

*SD, Standard deviation; BDT, Bangladeshi Taka*.

* *Fisher's Exact test*.

### Financial Concerns and Their Correlates

Most participants reported their household income was decreased due to the impact of COVID-19 (96.3%). Household income decreases due to the impact of COVID-19 were related to (i) being female vs. male (98.5 vs. 94.5%, *p* = 0.039), (ii) having had primary vs. no formal education (97.8 vs. 91.1%, *p* = 0.010), (iii) having had lost jobs vs. not (97.0 vs. 40.0%, *p* < 0.001), (iv) having had experienced food scarcity due to COVID-19 vs. not (97.4 vs. 73.7%, *p* < 0.001), and (v) having higher vs. lower depression scores (7.2 ± 4.4 vs. 5.0 ± 5.0, *p* = 0.05).

### Associations With Depression and PTSD

**Figure 2:**
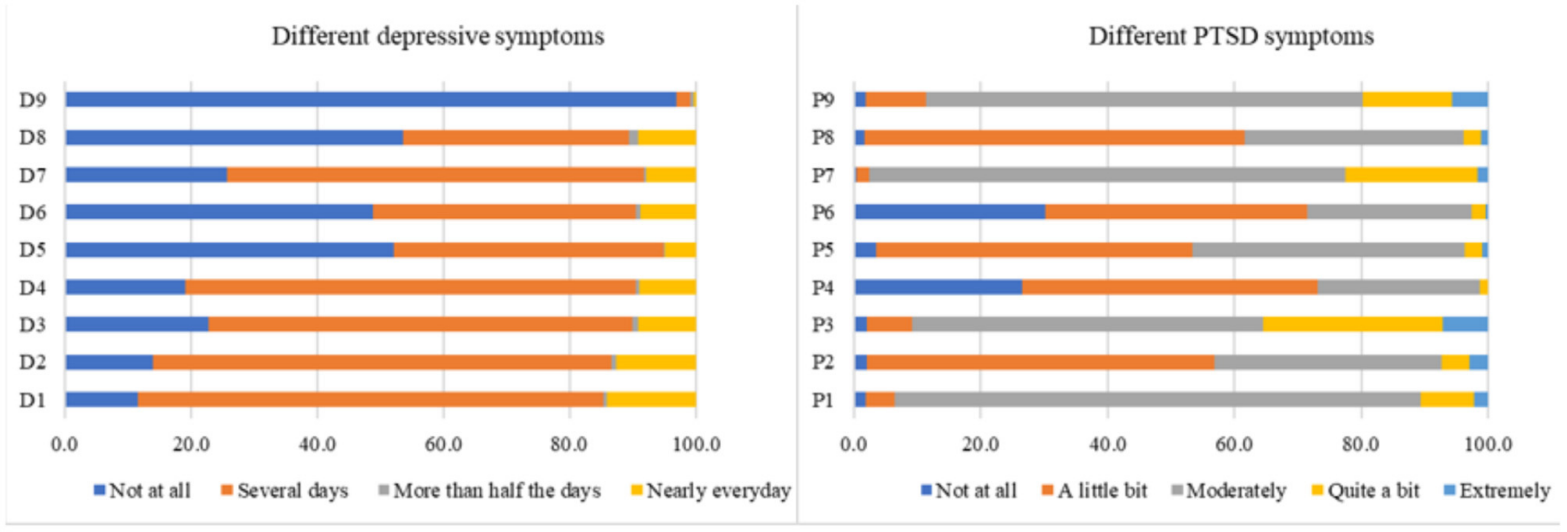
Symptoms of depression and PTSD. D1, Little interest or pleasure in doing things; D2, Feeling down, depressed, or hopeless; D3, Trouble falling or staying asleep, or sleeping too much; D4, Feeling tired or having little energy; D5, Poor appetite or overeating; D6, Feeling bad about yourself or that you are a failure or have let yourself or your family down; D7, Trouble concentrating on things, such as reading the newspaper or watching television; D8, Moving or speaking so slowly that other people could have noticed. Or the opposite being so fidgety or restless that you have been moving around a lot more than usual; D9, Thoughts that you would be better off dead, or of hurting yourself; P1, Having “flashbacks”, that is, you suddenly acted or felt as if a stressful experience from the past was happening all over again (for example, you re-experienced parts of a stressful experience by seeing, hearing, smelling, or physically feeling parts of the experience); P2, Feeling very emotionally upset when something reminded you of a stressful experience; P3, Trying to avoid thoughts, feelings, or physical sensations that reminded you of a stressful experience; P4, Thinking that a stressful event happened because you or someone else (who didn't directly harm you) did something wrong or didn't do everything possible to prevent it, or because of something about you; P5, Having a very negative emotional state (for example, you were experiencing lots of fear, anger, guilt, shame, or horror) after a stressful experience; P6, Losing interest in activities you used to enjoy before having a stressful experience; P7, Being “super alert”, on guard, or constantly on the lookout for danger; P8, Feeling jumpy or easily startled when you hear an unexpected noise; and P9, Being extremely irritable or angry to the point where you yelled at other people, got into fights, or destroyed things.

The mean scores of depression and PTSD were 7.1 ± 4.4 (out of 27) and 15.1 ± 3.4 (out of 36), respectively. Features related to depression and PTSD are presented in Figure 2. Of note, depression and PTSD were significantly and positively correlated with each other (Pearson *r* = 0.34; *p* < 0.001).

Table 2 summarizes a multiple regression analysis statistically predicting depression and PTSD. In Model 1, all examined variables were entered together to examine associations with depression symptoms. Higher depression scores were positively associated with female gender (β = 0.19; *p* = 0.035), joblessness (β = 0.11; *p* = 0.043), being divorced (β = 0.20; *p* < 0.001), living in joint family (β = 0.13; *p* = 0.01), excessive sleep (>9/day) (β = 0.09; *p* = 0.045), and smoking (β = 0.14; *p* = 0.008). The regression Model 1 predicted 17% of the variance in depression scores (*F*_(20, 414)_ = 5.53, *p* < 0.001).

**Table 2:** Multiple regression analysis predicting depression and PTSD.

**Characteristics**	**Depression** ^a^							**PTSD** ^b^						
	***Mean***	***(SD)***	***B***	***95% CI***	**β**	***SE***	***p-value***	***Mean***	***(SD)***	***B***	***95% CI***	**β**	***SE***	***p-value***
**Gender**														
Male	6.4	(4.0)			^†^			14.9	(3.7)				^†^	
Female	8.1	(4.7)	1.68	(0.12–3.25)	0.19	0.80	0.035	15.4	(3.0)	0.21	(−1.04–1.46)	0.64	0.03	0.744
**Age**														
18–40 years	6.6	(4.0)			^†^			15.1	(3.1)				^†^	
>40 years	7.6	(4.7)	0.50	(−0.36–1.35)	0.06	0.43	0.253	15.2	(3.6)	0.02	(−0.66–0.70)	0.35	0.00	0.958
**Education**														
Secondary level (6–10 grades)	7.5	(4.4)			^†^			15.3	(3.6)				^†^	
No formal education	7.6	(5.9)	−0.76	(−2.36–0.85)	−0.06	0.82	0.355	15.6	(5.5)	−0.10	(−1.39–1.19)	0.66	−0.01	0.882
Primary level (1–5 grades)	7.0	(4.1)	−1.14	(−2.38–0.11)	−0.11	0.63	0.073	15.0	(2.8)	−0.50	(−1.50–0.50)	0.51	−0.06	0.326
**Occupation**														
Housewife	8.6	(5.3)			^†^			15.1	(2.8)				^†^	
Workers	6.9	(3.6)	−1.07	(−2.76–0.62)	−0.10	0.86	0.214	15.5	(2.9)	0.03	(−1.32–1.38)	0.69	0.00	0.969
Day laborer	6.0	(5.0)	−0.65	(−3.23–1.93)	−0.03	1.31	0.620	14.5	(6.2)	−0.63	(−2.69–1.44)	1.05	−0.04	0.552
Rickshaw puller	6.9	(3.5)	0.36	(−1.73–2.44)	0.03	1.06	0.737	15.4	(3.4)	0.85	(−0.82–2.52)	0.85	0.10	0.318
Jobless	11.3	(5.8)	2.12	(0.07–4.16)	0.11	1.04	0.043	16.2	(4.2)	0.34	(−1.30–1.98)	0.83	0.02	0.683
Small shop keeping	6.3	(4.2)	−0.18	(−2.00–1.64)	−0.02	0.92	0.845	14.8	(2.9)	0.15	(−1.31–1.60)	0.74	0.02	0.844
Others	6.4	(4.7)	−0.21	(−2.5–2.08)	−0.01	1.17	0.858	14.1	(3.3)	−0.78	(−2.62–1.05)	0.93	−0.06	0.401
**Marital status**														
Married	6.7	(3.9)			^†^			15.0	(3.4)				^†^	
Unmarried	6.3	(3.6)	−0.54	(−2.54–1.46)	−0.03	1.02	0.598	16.5	(4.2)	1.50	(−0.11–3.10)	0.82	0.09	0.067
Divorced	12.1	(6.9)	3.31	(1.61–5.02)	0.20	0.87	<0.001	16.1	(3.0)	0.57	(−0.80–1.93)	0.69	0.05	0.416
**Family type**														
Nuclear	6.7	(4.0)			^†^			15.0	(3.3)				^†^	
Joint	10.5	(6.3)	1.87	(0.45–3.29)	0.13	0.72	0.010	16.4	(4.0)	1.02	(−0.12–2.16)	0.58	0.09	0.080
**Monthly family income**														
>10,000 BDT	7.0	(4.5)			^†^			14.6	(2.9)				^†^	
≤ 10,000 BDT	7.2	(4.4)	0.74	(−0.26–1.74)	0.08	0.51	0.144	15.6	(3.7)	1.18	(0.38–1.98)	0.41	0.17	0.004
**Sleep status**														
7–9 h	6.9	(4.5)			^†^			15.4	(3.7)				^†^	
<7 h	7.5	(4.3)	0.73	(−0.12–1.57)	0.08	0.43	0.090	14.6	(2.7)	−0.82	(−1.49–0.15)	0.34	−0.11	0.017
>9 h	10.7	(4.6)	3.53	(0.08–6.97)	0.09	1.75	0.045	18.0	(1.9)	3.19	(0.44–5.95)	1.40	0.11	0.023
**Tobacco smoking**														
No	7.1	(4.6)			^†^			15.2	(3.5)				^†^	
Yes	7.3	(3.8)	1.50	(0.39–2.61)	0.14	0.56	0.008	15.0	(2.9)	0.03	(−0.86–0.92)	0.45	0.00	0.946
**Job loss due to COVID-19**														
No	6.5	(6.1)			^†^			12.6	(6.6)				^†^	
Yes	7.2	(4.3)	−0.23	(−2.3–1.84)	−0.01	1.05	0.829	15.3	(3.1)	2.18	(0.52–3.84)	0.84	0.13	0.010
**Experiencing food scarcity due** **to the COVID-19 pandemic**														
No	2.8	(3.0)			^†^			9.6	(7.3)				^†^	
Yes	7.2	(4.4)	1.91	(−2.06–5.88)	0.05	2.02	0.345	15.2	(3.3)	4.38	(1.20–7.56)	1.62	0.14	0.007
**Household income decreases due** **to the COVID-19 pandemic**														
No	5.0	(5.0)			^†^			13.7	(7.2)				^†^	
Yes	7.2	(4.4)	1.48	(−0.75–3.71)	0.06	1.13	0.193	15.2	(3.2)	0.36	(−1.43–2.14)	0.91	0.02	0.694

*SD, Standard deviation; B, Unstandardized regression coefficient; CI, Confidence interval; SE, Standard error; β, Standardized regression coefficient; BDT, Bangladeshi Taka*.

† *Reference category*.

a *Model summery (Depression): F_(20, 414)_ = 5.53, p < 0.001, $R_{Adj}^{2}$ = 0.17*.

b *Model summery (PTSD): F_(20, 414)_ = 3.33, p < 0.001, $R_{Adj}^{2}$ = 0.10*.

Next, all measures were entered together to examine associations with PTSD symptoms in Model 2. Higher PTSD scores were positively associated with monthly incomes ≤ 10,000 BDT (β = 0.17; *p* = 0.004), excessive sleep (>9 h/day) (β = 0.11; *p* = 0.023), joblessness due to the COVID-19 pandemic (β = 0.13; *p* = 0.010), and experiencing food scarcity due to the COVID-19 pandemic (β = 0.14; *p* = 0.007); and less sleep (<7h/day) was negatively associated with PTSD scores (β = −0.11; *p* = 0.017). The regression Model 2 predicted 10% of the variance in PTSD scores (*F*_(20, 414)_ = 3.33, *p* < 0.001).

## Discussion

The COVID-19 pandemic has exerted psychological and financial impacts on many people (Banna et al., 2020; Bodrud-Doza et al., 2020; Islam et al., 2020c; Zubayer et al., 2020). Among urban-dwelling individuals worldwide, the pandemic has particularly impacted impoverished residents compared to others, especially those living in low- and middle-income countries (Tampe, 2020). The present study investigated financial hardships and, the symptoms of depression and PTSD symptoms among impoverished urban residents in Dhaka, Bangladesh during the COVID-19 pandemic. Notably, the vast majority of individuals (> 95%) experienced decreased household incomes, job losses and food insecurity during the COVID-19 pandemic. These stress-eliciting experiences suggest that biocentric strategies that target stress and increase coping (e.g., mindfulness-based stress reduction) coupled with changing environmental contexts (e.g., addressing crowded living situations and poverty) are needed to improve the health of impoverished inhabitants of Dhaka. As there have been no prior studies in Bangladesh investigating financial hardships along with mental health among this marginalized group, the present findings have been placed in the context of findings from prior studies undertaken in different regions and involving different populations.

The COVID-19 pandemic has had a major impact on the country's economy and on individuals, apart from its impacts on the national health situation (Bodrud-Doza et al., 2020). Due to the current pandemic, many individuals or families have lost their sources of income (The World Bank, 2020). In the present study, 96.3% of respondents reported a decrease in their household income due to the impact of the COVID-19 pandemic. This percentage is higher than those in Vietnam (66.9%) (Tran et al., 2020) and India (45.6%) (Keelery, 2020). The disparity could be attributable to differences in economic structures and major markets between countries. The economy of Bangladesh has heavily relied on ready-made garments and foreign remittance, both impacted substantially by the COVID-19 pandemic (Amit, 2020). In this study, the decreased household income was associated with being female, having a primary education level, having lost jobs, having experienced food scarcity and having experienced more symptoms of depression. Household income is reduced more among females than males due to the impact of the COVID-19 pandemic resonates with prior findings (Rudkin, 1993). A primary education level was associated with decreased household income during the COVID-19 pandemic, possibly owing to these individuals being in unskilled and “disposable” employment. In addition, decreased household income during the COVID-19 pandemic was linked to job losses, food scarcity and depression. Several of these factors may relate to periods of lockdown or closure of businesses following the requirement of strict spatial distancing in Bangladesh, and longer-term and longitudinal studies to investigate this possibility are needed.

In the present study, the regression analysis exhibited that being female, experiencing joblessness, being divorced, living in a joint family, sleeping excessively (>9 h/day), and smoking were associated with higher depression scores. The finding of women reported higher depression scores than men is consistent with previous reports (Van Droogenbroeck et al., 2018; Hossain et al., 2019; Islam et al., 2020f). Females may experience increased emotional vulnerability and suffer more from stressors related to negative psychological effects, such as the death of friends or family, and these factors may hold relevance during a pandemic (Matheson et al., 2014; Van Droogenbroeck et al., 2018).

Participants with joblessness attributable to the COVID-19 pandemic experienced more depression, consistent with prior studies (Mandal et al., 2011; Stolove et al., 2017). The COVID-19 pandemic has generated job loss/displacement, resulting in decreased incomes (Tran et al., 2020), and these events may contribute to depression.

Being divorced was associated with depression in the present study, consistent with previous reports (Sbarra et al., 2014). This association may reflect loneliness, isolation, and other social problems that may be experienced in a more solitary fashion when divorced and may be particularly impactful during the pandemic (Saltzman et al., 2020). The present study also observed an association between depression and living in joint families, in line with prior findings (Mishra et al., 2018); however, the finding is distinct from those in a prior Bangladeshi study (Islam et al., 2020f) reporting no relationship between family type and depression. In contrast, prior data have linked residing in nuclear families to depression (Taqui et al., 2007). These differences may reflect certain circumstances relating to impoverished living settings during a pandemic that speculatively may include financial hardships related to greater monthly household costs, job displacements, food scarcity or other factors. These considerations warrant further investigation.

Excessive sleep (>9 h/day) was associated with depression. This finding aligns with a prior Bangladeshi longitudinal study (Hossain et al., 2019) and reviewed findings (Lovato and Gradisar, 2014) but appears to contrast with prior Bangladeshi reports (Anjum et al., 2019; Islam et al., 2020a) that observed no association between sleeping hours and depression. The extent to which the findings might relate to longer sleep of poorer quality given crowded living situations or other factors warrants additional study. The present findings also linked smoking to depression, consistent with prior reports in Bangladesh (Islam et al., 2020f, 2021d; Tasnim et al., 2021), global findings from 48 low- and middle-income countries (Stubbs et al., 2018) and a systematic review (Fluharty et al., 2017).

In regression analyses, higher PTSD scores were associated with monthly incomes ≤ 10,000 BDT, excessive sleep (>9 h/day), job loss due to the COVID-19 pandemic and food scarcity due to the COVID-19 pandemic. This study revealed no gender-related difference relating to PTSD symptoms, which differs from a recent study from China during the COVID-19 pandemic that reported higher PTSD symptomatology among females compared to males (Liu et al., 2020). The extent to which this difference may reflect cultural differences or distinct populations warrants additional study.

Lower income (≤ 10,000 BDT ≈ ≤ 118 US$ per month) and sleep disturbances (increased) were associated with more severe PTSD symptomatology, in line with previous reports (Maher et al., 2006; Parto et al., 2011). A scoping review also concluded that individuals who suffer from PTSD have sleep disturbances (Magnavita and Garbarino, 2017). The seemingly protective effect of less sleep warrants further investigation.

The present study also indicated that job loss and food insecurity due to the COVID-19 pandemic were associated with more severe PTSD symptomatology. Spatial distancing and lockdown measures in conjunction with living in close quarters are factors that speculatively may generate PTSD symptoms. Other factors, including disruptions to everyday life and routines, financial hardships, job losses, and diminished social support, may also contribute to PTSD symptoms (Boyraz and Legros, 2020; Islam et al., 2020d). Low-income communities may be at particular risk of developing PTSD as impoverished urban residents may experience more trauma than some other groups living in more well-developed areas (Boyraz and Legros, 2020). A dense population, congested living accommodations and lower incomes warrant consideration in the development and addressing of PTSD symptomatology. PTSD develops when symptoms from a psychological trauma disrupt daily functioning and last for over a month. PTSD symptoms may persist for decades if not treated (Bo et al., 2020). Therefore, the present study suggests the need for effective interventions, including outreach efforts, psychopharmacological treatments and behavioral therapies (including mind-body interventions), and addressing of traumatic living situations and life experiences (Horesh and Brown, 2020).

Of note, the largely anthropocentric measures collected and modeled in the current study accounted for relatively small amounts of variance in relation to mental health measures. These findings suggest that future models should consider additional factors. In this process, biocentric factors may be important to consider. The current study focused on impoverished urban-dwelling individuals in Dhaka city during the COVID-19 pandemic. Considering the pandemic from a larger focus, including with respect to how cities interact with nature and respond during emergencies like the pandemic, will be important moving forward (de Leeuw, 2020). Cities have been described as important governing bodies in responses to crises like the COVID-19 pandemic, but have long struggled with substantial inequities, including those between humans and other species. Citizens in cities have often been impacted disproportionately by disease burden, and this appears true during the COVID-19 pandemic. To address current challenges, it will be important to approach city planning using biocentric strategies in response to and when recovering from the COVID-19 pandemic. Such approaches should be grounded in models that consider biocentric relationships between people and themselves, other humans and other organisms in nature (Stueck, 2021). As other pandemics are likely to occur in the future, addressing these concerns in a timely fashion is important.

### Limitations

There are some limitations that warrant discussion. This study was cross-sectional; thus, it is not possible to make causal inferences. Future longitudinal studies are needed. The findings may not generalize to other impoverished urban residents beyond Dhaka, Bangladesh. Future larger-scale studies involving other jurisdictions are warranted. The study gathered a limited number of COVID-19-related assessments. Future studies should examine additional relevant COVID-19-related domains using validated questions. Further, as decreases in household income were highly prevalent, ceiling effects may have influenced findings. Although the regression models considered a large number of variables, they captured relatively small amounts of the variances.

## Conclusions

Marginalized communities like impoverished urban residents have been greatly impacted amid the COVID-19 pandemic perhaps given their dense populations, congested accommodations and low incomes. In the current study, most impoverished urban residents reported decreased household incomes due to the COVID-19 pandemic. The findings indicated that depression symptoms were associated with being female, joblessness, being divorced, living in a joint family, excessive sleep and smoking. Low incomes, excessive sleep, joblessness and food scarcity were associated with PTSD symptoms. In contrast, less sleep appeared protective against PTSD. There is a crucial need for a thorough evaluation of the effects of the COVID-19 pandemic on various groups over the next decade, which will inform the government of the need to introduce effective policies to ease the economic and psychological pain of vulnerable communities. Considering a biocentric perspective and strategies that consider mind-body relationships and ethical attitudes toward the environment (e.g., addressing impoverished living situations) should help promote individual and public health. Public health initiatives, in particular mental health services, should be introduced to mitigate the psychological effects of the pandemic on impoverished urban residents and other vulnerable populations.

## Data Availability Statement

The raw data supporting the conclusions of this article will be made available by the authors, without undue reservation.

## Ethics Statement

The studies involving human participants were reviewed and approved by the ethical review board of Jahangirnagar University [Ref. No: BBEC, JU/M 2020/COVID-19/(8)5]. The patients/participants provided their written informed consent to participate in this study.

## Author Contributions

MI, ME, and SH: conceptualization and investigation. MI: methodology and formal analysis. ME: resources. SH: supervision. MI, MR, and RB: writing—original draft preparation. NS, SH, MH, MS, LS, and MP: writing—review and editing. MP: critical revision. All authors have read and approved the final manuscript.

## Conflict of Interest

The authors declare that the research was conducted in the absence of any commercial or financial relationships that could be construed as a potential conflict of interest.

## Publisher's Note

All claims expressed in this article are solely those of the authors and do not necessarily represent those of their affiliated organizations, or those of the publisher, the editors and the reviewers. Any product that may be evaluated in this article, or claim that may be made by its manufacturer, is not guaranteed or endorsed by the publisher.
